# A Case of Prolonged Wernicke’s Encephalopathy After Treatment With IV Thiamine Due to the Subsequent Development of Refeeding Syndrome

**DOI:** 10.7759/cureus.65178

**Published:** 2024-07-23

**Authors:** Sally Namboodiri, Sanjay Rao

**Affiliations:** 1 Medicine, Louis Stokes Cleveland VA Medical Center, Cleveland, USA; 2 Radiology, Brigham and Women's Hospital, Harvard Medical School, Boston, USA

**Keywords:** refeeding syndrome, thiamine or vitamin b1 deficiency, alcohol use disorder (aud), persistent hypokalemia, cognitive disorders, oculomotor disorders, nutritional deficiency, neurologic exam, gait disturbance, persistent delirium

## Abstract

Wernicke’s encephalopathy (WE) is a rare, life-threatening condition in which thiamine deficiency causes dysfunction of the Kreb’s cycle, accumulation of lactic acid in the brain tissues, and irreversible cognitive impairment. Prompt treatment with IV thiamine can reverse the process. The classic Wernicke’s triad of ataxia, memory issues, and ocular abnormalities is not often present. Caine’s criteria, which requires two of the following: dietary deficiencies, ocular abnormalities, altered cognition or mental status, and cerebellar dysfunction, is highly sensitive and specific for Wernicke’s diagnosis, especially in patients with alcohol use disorder. Refeeding syndrome (RS) has similar risk factors to WE, including disease states that lead to malnutrition. Patients with RS develop WE due to thiamine depletion that occurs when oral nutrition is reinitiated after a period of poor oral intake. We present a patient with initially undetected WE who developed RS after the initiation of treatment with IV thiamine. RS prolonged the neurologic symptoms of WE and led to an extended hospital stay and significant physical debility. In our patient, WE preceded RS instead of occurring as a consequence of it. The case highlights that if one of these disorders is present, the other may not be far behind. When WE precedes RS, prolonged treatment with IV thiamine may be warranted until the symptoms of both disorders resolve.

## Introduction

Wernicke’s encephalopathy (WE) is an often-unrecognized cause of delirium in hospitalized patients. Its prevalence is between 1% and 3%, but this data mainly comes from autopsy studies because the majority of cases go undiagnosed [1]. WE results from a deficiency of thiamine (vitamin B1), which is a critical coenzyme in the Krebs cycle. If left untreated, WE can lead to death in greater than 20% of cases and dementia, or Korsakoff’s syndrome, in more than 80% of surviving patients [2]. Risk factors for WE include alcohol use, malabsorption, bariatric surgery, malignancy, hyperemesis gravidarum, anorexia, or other causes of extreme weight loss or malnutrition [3]. In patients with possible WE, prompt treatment with IV thiamine is indicated as oral thiamine may be poorly absorbed in patients who are malnourished. Because WE is a medical emergency, treatment should be started immediately upon clinical suspicion.

Like WE, refeeding syndrome (RS) is a life-threatening disorder in malnourished patients that is often overlooked. It is defined as potentially fatal shifts in electrolytes and fluid that occur when malnourished patients start receiving nutrition [4]. Since randomized controlled trials on RS are limited, there is no consensus regarding definitions or treatment approaches, and the prevalence of RS is not known [5]. Due to thiamine’s role in glucose-dependent energy production, its levels are also depleted during RS. For patients with RS, electrolyte and fluid shifts, along with thiamine depletion, can occur within two to five days of refeeding and may lead to heart failure, fatal arrhythmias, musculoskeletal manifestations (i.e., rhabdomyolysis, weakness, myalgias, fatigue, muscle twitching), and WE [6]. A systematic review of the role of thiamine supplementation in the treatment of RS found that while studies had small sample sizes and lacked generalizability, many patients with RS who developed WE improved clinically with thiamine supplementation. Despite the lack of randomized controlled trials, the authors concluded that thiamine has a role in the symptoms of RS, thiamine supplementation is not harmful, and it may be helpful in the treatment of RS [7]. However, in this case report, we present a patient with a delayed diagnosis of WE who developed RS a few days after initiation of IV thiamine; as a result, his WE symptoms took weeks to resolve and led to a prolonged hospital stay and significant physical debility.

## Case presentation

A 62-year-old male with a history of post-traumatic stress disorder on escitalopram 20 mg daily, alcohol use disorder in remission for four months, hypokalemia on potassium chloride 20 milliequivalents (meq) daily, and tobacco use of 1 pack of cigarettes per day for 40 years presented to the emergency department (ED) with a 60-pound weight loss, lack of appetite, nausea, and constipation for two months. He had recently been released from a seven-year prison sentence and was living in a group home for four months. He denied abdominal pain, depression, trouble chewing or swallowing, vomiting, fever, or chills. He complained of trouble walking due to weakness but denied falls. His daughter was concerned that his memory was worsening for the past one month as he had missed some medical appointments and misplaced his medications. On physical exam, he was afebrile, with blood pressure of 120/80 mmHg, pulse of 58 beats per minute, oxygen saturation of 95% on room air, a weight of 138 pounds, down from 168 pounds three months prior, with a body mass index of 22. The exam was remarkable for mild diffuse abdominal tenderness. He was alert and oriented to person, place, and time with normal speech. Lab testing in the ED showed serum potassium of 2.9 mmol/L (range: 3.5-5.0), magnesium 2.0 mg/dl (range: 1.8-2.4), and normal complete blood count. Non-contrast CT scan of the abdomen demonstrated no acute abnormality and the non-contrast CT head demonstrated periventricular white matter changes. His potassium was repleted orally. He was admitted to the ward with a diagnosis of unintentional weight loss and initial plans were to pursue colonoscopy and upper endoscopy. He was maintained on low-dose IV fluids due to his poor appetite and a nutrition consult was obtained.

**Figure 1:**
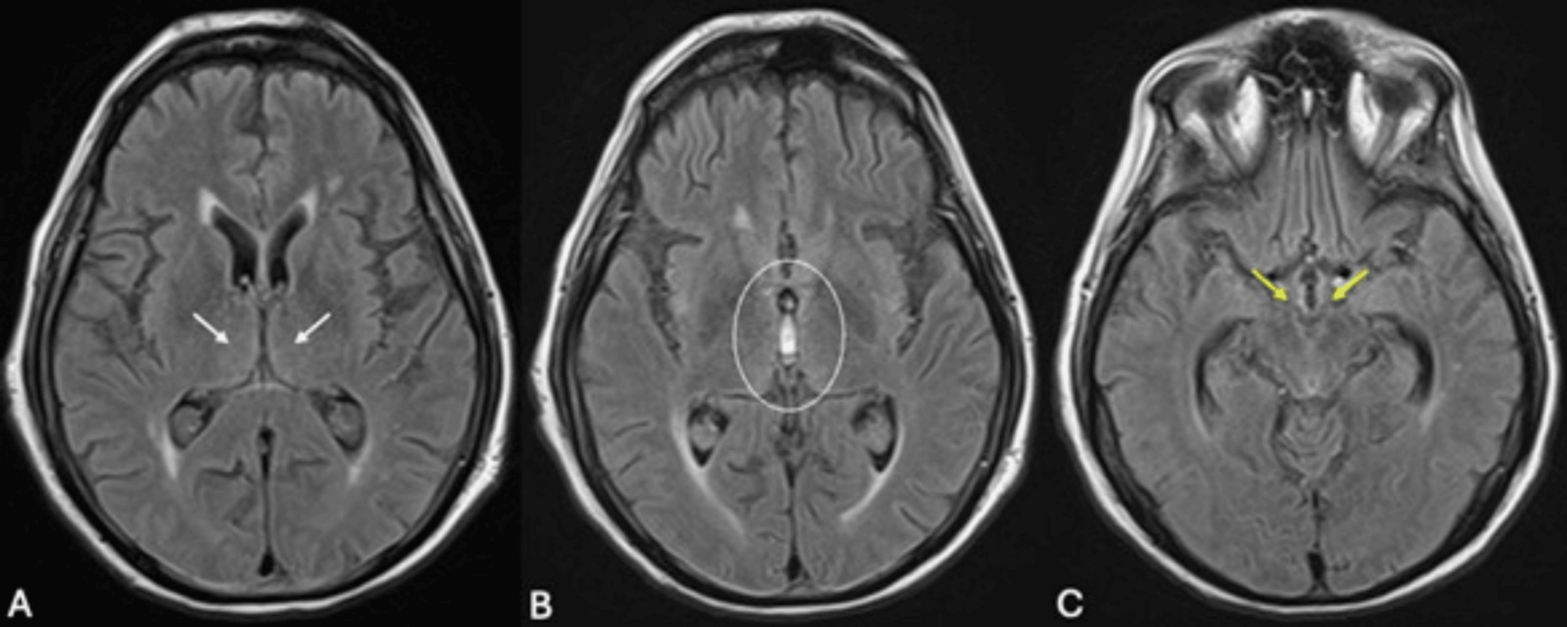
Fluid-attenuated inversion recovery (FLAIR) brain magnetic resonance (MR) images at the level of the (a) thalami (white arrows), (b) third ventricle (circle), and (c) mammillary bodies (yellow arrows) showing a normal MR signal in these regions. These areas may show increased FLAIR signal in cases of Wernicke’s encephalopathy or may be normal, as in this case.

On hospital day four, our patient fell out of bed and developed confusion, agitation, and visual hallucinations. He was diagnosed with acute delirium. Lab work showed a normal white blood cell count and normal electrolytes. Repeat non-contrast CT head showed no acute changes and an infectious workup was negative. The patient was noted to have constipation and urinary retention; a urinary catheter was placed and a bowel regimen was initiated. He remained hemodynamically stable but continued to have intermittent hallucinations and confusion. A psychiatry consult recommended low-dose antipsychotics for the hallucinations, but his family declined such medications. On hospital day 14, a neurologic exam revealed horizontal nystagmus and cerebellar ataxia. WE was suspected and he was started on IV thiamine 500 mg three times a day for two days, followed by 250 mg IV daily for five days, followed by oral thiamine 200 mg daily. An MRI brain showed no evidence of typical WE changes in the thalami, third ventricle, or mammillary bodies (Figure 1).

**Figure 2:**
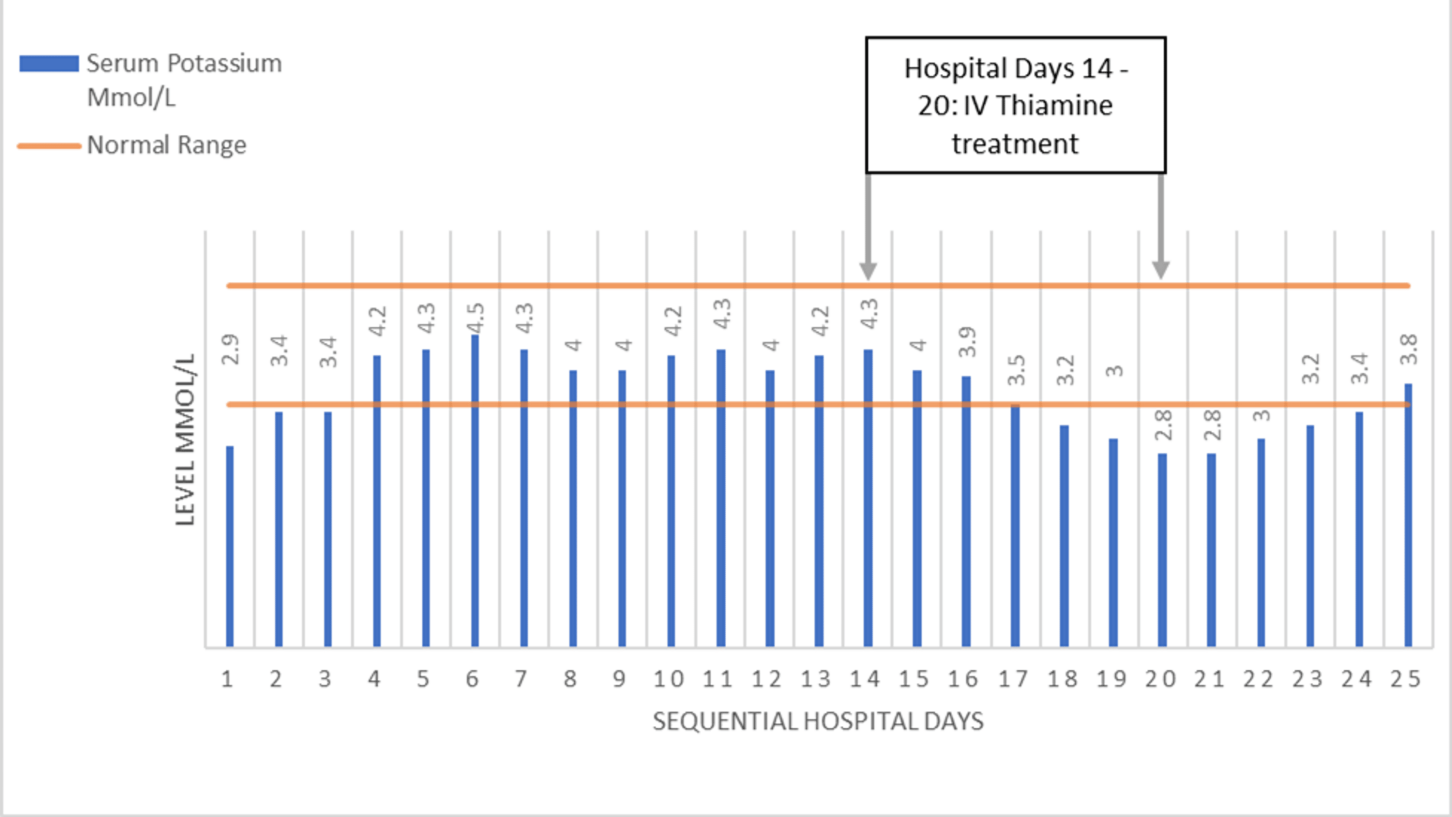
Serum potassium trend during hospital admission (serum reference range: 3.5-5.2 mmol/l). In addition, on hospital day 2, urine potassium was elevated at 25 mmol/L, and by hospital day 19, after IV thiamine was started, it decreased to 10 mmo/l.

After the initiation of IV thiamine, the patient’s agitation, confusion, and hallucinations initially improved. His appetite increased and his diet was advanced from nothing by mouth to an unrestricted regular diet. By hospital day 18, four days after initiating IV thiamine, his potassium began to drop (Figure 2) and his confusion and hallucinations recurred. In addition, he developed diffuse muscle twitching and increased muscle weakness. His horizontal nystagmus persisted. The potassium remained low for several days despite oral repletion with potassium chloride 20 to 40 meq daily; his magnesium was normal. His urinary potassium, which was elevated on admission, was reduced by hospital day 19 (Figure 2). A neurology consult recommended a repeat MRI brain, which was unchanged from the prior scan, and an electroencephalogram, which showed diffuse slowing and no seizure activity. In hindsight, we think the patient developed RS with persistent hypokalemia after initiation of feeding. By day 25, his hypokalemia resolved, and over the following several days, his mental status and neurologic exam slowly returned to baseline. Despite physical therapy, he had a persistent weakness with the inability to stand or walk unaided. He was discharged to a long-term care facility on hospital day 35.

## Discussion

WE is an often missed, life-threatening disorder. It is usually not considered in the differential diagnosis of delirium in part because thiamine deficiency is not included as an etiology in most guidelines on delirium [8]. WE is also underrecognized because the classic triad described by Wernicke of altered mental status, ataxic gait, and ophthalmoplegia, is rarely present; in one study, the triad occurred in fewer than 20% of cases and up to 19% of patients had none of these signs [9]. The Caine criteria, published in 1997, requires two of the four following criteria to diagnose WE: dietary deficiency, oculomotor abnormalities, cerebellar dysfunction, and either altered mental status or mild memory impairment. These criteria were studied in patients with alcohol use disorder, where they had a sensitivity of 85% and specificity of 100% [10]. Caine criteria, which includes elements of the classic triad, but adds the very important element of malnutrition, increases the likelihood that WE would be considered, especially since only two out of four elements are required for the diagnosis. Our patient had all four Cain criteria symptoms and may have been diagnosed with WE earlier in his hospital course if a neurologic exam had been performed on admission that revealed cerebellar and oculomotor abnormalities. Given his family’s concern about his memory, a mini mental status exam on admission may have revealed cognitive impairment that would further increase suspicion of WE and prompted early treatment with IV thiamine prior to his development of prolonged delirium. Our patient was started on IV thiamine by the hospital on day 14 once WE was suspected. Although there is no consensus on the dose, frequency, or duration of treatment with IV thiamine, many guidelines recommend 500 mg IV three times a day for two days followed by 250 mg for up to five days, followed by daily oral thiamine [11].

WE is a clinical diagnosis; a normal blood level of thiamine does not rule out WE as it does may not accurately reflect the level in the brain [12]. MRI of the brain in WE patients may show changes in the mammillary bodies, thalami, tectal plate, and periaqueductal area, and while the specificity of MRI for WE is high at 93%, the sensitivity is much lower at 53% [8], so it cannot be used to rule out the diagnosis. The fact that our patient’s MRI brain lacked WE change highlights that clinicians must have a high index of suspicion for WE on a clinical basis and should not delay life-saving treatment by relying on imaging that may be potentially unhelpful [13].

On admission, our patient’s potassium was low, likely because he misplaced his oral supplement prior to admission, but it promptly returned to normal on the oral supplement. His high urine potassium on admission in the setting of low serum potassium suggests renal potassium wasting. Thiamine deficiency leads to impairment of the Krebs cycle’s adenosine triphosphate (ATP) synthesis; similarly, it may cause renal tubular dysfunction due to impairment of the tubular transport system, which is ATP-dependent [14,15], thus leading to electrolyte abnormalities. Case studies show that thiamine administration can lead to the resolution of renal tubular dysfunction and the normalization of electrolytes [16]. Our patient’s renal potassium wasting resolved as evidenced by a reduction in his urine potassium level after he was given IV thiamine. His longstanding hypokalemia may have been a sign of chronic, mild thiamine deficiency with renal potassium wasting that eventually became severe, leading to his WE presentation.

Our patient was also at high risk for RS due to his significant weight loss, hypokalemia, and history of alcohol use disorder. Table 1 summarizes the National Institute for Health and Clinical Excellence (NICE) guidelines to identify patients at risk for RS [6]. RS has similar risk factors as WE and includes patients with weight loss, poor nutrition, and alcohol use disorder. Although there is no overall consensus on the management of RS, NICE, and the American Society for Parenteral and Enteral Nutrition (ASPEN) have put out similar management recommendations. ASPEN proposes checking phosphorus, potassium, and magnesium and repleting low levels prior to reinitiating oral nutrition in high-risk patients with severe electrolyte abnormalities. Electrolytes should be checked twice a day for the first three days and once in the normal range, patients should receive 100-150 grams of carbohydrate (10 to 20 kcal/kg) in the first 24 hours and increase the amount by 33% every one to two days until the nutrition goal is met [17]. ASPEN and NICE guidelines both recommend repleting thiamine prior to initiating feeds to prevent further depletion of thiamine during RS [6,17]. Although phosphate deficiency has been the electrolyte abnormality most widely associated with RS, ASPEN feels this is due to definition bias and that potassium and magnesium levels are just as likely to be affected [17]. Several clues suggested the development of RS: 1) the timing of the hypokalemia, which occurred a few days after the patient started to eat and did not respond to oral supplementation (Figure 2); 2) a low urine potassium level (Figure 2), likely from intracellular movement of potassium; 3) the development of muscle twitching and worsening weakness, both presumably from hypokalemia; and 4) persistent WE symptoms despite treatment with IV thiamine. His prolonged nystagmus and delirium suggested further depletion of thiamine from RS, especially since his thiamine was changed from IV to oral form while he still had low serum potassium levels (Figure 2).

**Table 1:** Adapted from National Institute for Health and Clinical Excellence (NICE) guidelines to identify patients at risk for refeeding syndrome.

Patient has one or more of the following:	Or patient has two or more of the following:	
Body mass index < 16	Body mass index < 18.5	
Unintentional weight loss >15% in the past 3-6 months	Unintentional weight loss >10% in the past 3-6 months	
Little or no nutrition for >10 days	Little to no nutritional intake for >5 days	
	History of alcohol use disorder or use of insulin, chemotherapy, diuretics, or antacids	
Low levels of potassium, phosphate, or magnesium prior to feeding		

In their systematic review of the role of thiamine in the treatment of RS, Steiner et al. noted wide variability in the timeline of improvement of WE symptoms, with some studies noting immediate improvement, while others reported persistent nystagmus, ataxia, and memory deficits for days to months [7]. Similar to our patient, the patients in these studies with RS and persistent WE symptoms despite treatment with IV thiamine may have had WE prior to developing RS. In cases where WE clearly precedes RS, ongoing treatment with IV thiamine may be warranted until electrolyte abnormalities and clinical symptoms resolve.

## Conclusions

WE and RS are both life-threatening, underrecognized disorders that occur in patients with malnutrition, including those with alcohol use disorders. Patients with RS are at risk for WE and vice versa; many case reports describe patients with RS who develop WE from thiamine depletion. In our case, the patient had clear symptoms of WE early in his hospital course and later developed RS after initiation of IV thiamine.

RS, while a distinctly different disease process to WE, maybe its companion; when considering one diagnosis, we should be wary that the other one is not far behind. The risk of RS was overlooked in our patient, who started to improve clinically after initiation of IV thiamine for WE and began eating after a prolonged duration of poor oral intake. His unrestricted carbohydrate intake led to insulin spikes and potassium and thiamine depletion. RS caused his WE symptoms to persist despite a seven-day course of IV thiamine. In a review of the literature, some case reports describe rapid clinical improvement in patients with RS who receive IV thiamine, while others note prolonged neurologic symptoms despite thiamine treatment. The latter group of patients may be similar to our patient, where WE clearly preceded RS and led to a profound thiamine deficit that was not corrected with seven days of IV thiamine. In such patients, prolonged treatment with IV thiamine may be indicated until electrolyte abnormalities resolve and neurologic symptoms improve.
